# A protocol for a systematic review on intersectoral interventions to reduce non-communicable disease risk factors in African cities

**DOI:** 10.1016/j.puhip.2022.100251

**Published:** 2022-04-04

**Authors:** Ebele R.I. Mogo, Anna Brugulat-Panés, Lisa K. Micklesfield, Charles Ebikeme, Trish Muzenda, Louise Foley, Tolu Oni

**Affiliations:** a MRC Epidemiology Unit, Global Diet and Activity Research Group and Network, University of Cambridge, United Kingdom; b South African Medical Research Council, University of the Witwatersrand Developmental Pathways for Health Research Unit, Department of Paediatrics, Faculty of Health Sciences, University of the Witwatersrand, Johannesburg, South Africa; c Department of Health Policy, London School of Economics and Political Science, London, United Kingdom; d Research Initiative for Cities Health and Equity, School of Public Health and Family Medicine, University of Cape Town, Cape Town, 7925, South Africa

**Keywords:** Intersectoral interventions, Non-communicable diseases, Urban health, Population health, Africa

## Abstract

**Objectives:**

To present the protocol for a systematic review synthesising quantitative and qualitative evidence in academic and grey literature on intersectoral interventions to address non-communicable disease risk factors in urban Africa.

**Study design:**

This protocol is developed in accordance with the Preferred Reporting Items for Systematic Review and Meta-analyses Protocols guidelines. Databases to be searched include PubMed, Global Health, SCOPUS, and Web of Science. Grey literature will be sourced from Google, local, regional, and international agencies, colleagues within the GDAR network, international organisations such as the WHO and UN-Habitat, UNICEF's Child Friendly Cities Initiative, Partnership for Healthy Cities, WHO Alliance for Healthy Cities, the African Centre for Cities, as well as grey literature databases such as Greynet and Opengrey.

**Methods:**

We will include all quantitative and qualitative study designs that describe any initiatives to address non-communicable disease risk factors through intersectoral interventions, and those that describe associations between such interventions and behavioural health or wellbeing outcomes. We will also include health service interventions that have an intersectoral component and are focused on non-communicable disease prevention. Studies must have been conducted in African countries, published in the past 30 years, and contain primary or secondary data as well as an analysis of these data.

**Results:**

We will use the qualitative checklist and the cohort study checklist of the Critical Appraisal Skills Programme (CASP), to appraise the quality of each study included in this review. While the specific framework for data synthesis will be concluded after reviewing the extracted data, we anticipate using a parallel convergent method to synthesise the parallel strands of our study, as it involves analysing the qualitative and quantitative papers separately and then integrating them.

**Conclusions:**

This will be the first systematic review to explore intersectoral interventions to address non-communicable disease risk in African cities, thus filling a crucial gap in the literature. The findings of this study will be disseminated across global organisations whose mandates cut across non-communicable diseases prevention, health promotion and healthy urban development. These include but are not limited to the World Health Organization, UN-Habitat, the UN Interagency Task Force on Noncommunicable Diseases Prevention and Control and the NCD Global Coordination Mechanism. We also plan to disseminate our findings to national and provincial stakeholders such as local governments, Ministries of Health and grassroots organisations; intergovernmental organisations such as the African Development Bank, and local and international private foundations such as Dangote Foundation and the Gates Foundation. The pan-African scope of this study makes it eligible to serve as a regional body of work and a resource to inform future interventions, practices, and policies.

## 1 Introduction

Healthy city initiatives aim to create and/or improve the social and built aspects of environments, as well as the community resources they contain to promote the wellbeing of their residents [1]. Given that Africa is one of the fastest urbanising regions of the world, these initiatives are particularly necessary on the continent [2]. Projections indicate that Africa will triple its urban population from approximately 395 million people in 2010 to 1.339 billion people by 2050, the equivalent of a fifth of the world's projected urban population [1]. Some African cities with a current population of over 10 million inhabitants include Cairo (Egypt), Kinshasa (Democratic Republic of Congo), Lagos (Nigeria), Johannesburg-Pretoria (South Africa), Khartoum (Sudan), Nairobi (Kenya) and Accra (Ghana), while cities like Luanda (Angola) and Dar-es-salaam (Tanzania) are projected to join these ranks soon [3].

Inhabitants of cities enjoy an urban advantage, due to the increased access to employment and educational opportunities, social connections, and healthcare services [4]. Africa's mostly young urban population [5] presents an untapped opportunity for improved regional productivity and economic growth, if cities were conducive to their wellbeing. However, many residents and migrants to African cities live under informal conditions, in unsafe environments and without access to the economic opportunities and services that could improve their physical, mental, and social health [6]. Therefore, rather than experiencing an urban advantage, many residents of African cities face an urban penalty - with risk exposures that threaten their health [4]. Some of these exposures include rising intra-urban inequities and the double burden of infectious and non-communicable diseases (NCDs) which typically occur at younger ages than in developed countries [6].

Cities are becoming increasingly obesogenic, with a high prevalence of low nutrient energy-dense (LNED) foods, the marketing of unhealthy foods and substances, a lack of infrastructure for safe commuting and physical activity, and safe recreational facilities [[7], [8], [9]]. The obesogenic elements of African cities are interconnected and inextricable, and are influenced by the indiscriminate importation of health-harming commodities such as tobacco, alcohol [10] and ultra-processed foods. In the context of the stress associated with rising poverty and poorly planned urban development, these factors combined put residents at a higher risk for obesity and other NCDs [11].

Table 1 presents a picture of the urban health risks [[12], [13], [14], [15]] in Kenya, Cameroon and South Africa, countries represented in the Global Diet Activity and Research Network (GDAR) [16].

**Table 1:** Burden of obesity, physical inactivity, diabetes and urbanisation in selected African countries.

	Kenya		Cameroon		South Africa	
	Male	Female	Male	Female	Male	Female
Obesity in adults, %	3	9	5	14	15	39
Obesity in adolescents %	1	3	1	4	9	13
Physical inactivity %	13	16	20	34	26	48
Diabetes age-adjusted prevalence %	2.9	7.2	5.5			
Urban % 2018 (% urbanisation rate 2015-20)	27 (4.23)	56.4 (3.63)	66.4 (1.97)			
Informal settlement % of urban total 2014	56	37.8	23			

Intervening at critical leverage points in cities via coordinated intersectoral collaborations can improve public health across the life course, moving beyond solely risk factor aetiology towards a systems approach that embraces complexity and interrogates the dynamic linkages between aspects of urban life [17]. Such research would strengthen the basis for the causal inference of the impact of interventions on risk factors for poor health outcomes such as obesity.

Interventions such as initiatives to improve the walkability of neighbourhoods and increase access to healthy foods and spaces for leisure and physical activity should ideally consider: i) interlinked barriers to, and enablers of physical activity and healthy eating; ii) sociocultural perspectives on active living and healthy eating, as well as temporal-spatial built environment experiences of the community; iii) learnings from previous interventions in similar contexts; iv) an understanding of the current governance landscape of public and private sector planning and built environment initiatives; v) opportunities for shared learning through inter and intra-regional collaborations [18,19].

Interventions to address NCD risk in African cities must also address their distinct characteristics, of note, an abundance of informality and a preponderance of young people. While informal settlements are often considered illegal and unplanned, they remain a persistent feature of African cities [20]. Those living in informality are particularly vulnerable as they are least equipped to deal with health-harming urban exposures. Informally built environments add complexity as interventions to address obesity may not be implemented through formal regulated structures, like in high-income countries (HICs). This makes the adoption of interventions from HICs potentially ill-fitting in these contexts. Critical global goals such as the New Urban Agenda and the Sustainable Development Goals (SDGs) do acknowledge the need to improve, rather than eradicate, the conditions in informal settlements [21,22]. Resulting interventions are often limited in scope and tend to focus on access to water and sanitation. While this area of focus is critical for health, especially infectious disease prevention, there remains a gap in efforts to prevent and reduce NCDs in these contexts.

With a median age of 19.2 years, the population of Africa's cities is also noticeably young [5]. Adolescence, variably defined as 10–19 years [23] or 10–24 years [24], is a transitory phase of life within which people undergo physical, psychological and social changes and begin to gain independence outside of the family [25]. Key health behaviours are formed in this window [25]. Therefore, it is important to explore adolescence as another entry point for improving preconception health, lessening intergenerational NCD risk, and improving health across the life course.

### 1.1 Objectives

We describe the protocol for a systematic review which aims to synthesise existing primary quantitative and qualitative evidence in academic and grey literature on community and environmental interventions in urban Africa to address population health, with a focus on informal settings, reducing NCD risk, and adolescent-targeted initiatives.

The overall goal of the systematic review is to inform the discourse on developing population-level interventions to improve public health, and to reduce non-communicable disease risk in Africa.

Our specific objectives are to:

1) Synthesise peer-reviewed and grey literature on the development and implementation of initiatives to address NCD risk factors in African cities.
2) Describe and characterise these initiatives with respect to the targeted environment (formal or informal), setting (school, market etc.), health risk or behaviour focus, actors involved, and population of interest.
3) Identify evidence on the impact of these initiatives in general, on NCD prevention, and/or on adolescents.

Our research questions are as follows:

1) What healthy city initiatives have been documented in Africa?
2) What are the components of documented healthy city initiatives in Africa, specifically key actors and/or funders, target sectors, environments and settings, populations and outcomes of interest?
3) What is the impact of healthy city initiatives in Africa on health and wellbeing in general and NCD risk in particular?
4) What are the lessons learnt (barriers and facilitators) in the development and implementation of healthy city initiatives in Africa?
5) What are the gaps in the evidence base concerning healthy city initiatives that focus specifically on non-communicable disease prevention, adolescent wellbeing, and informal contexts in urban Africa?

## 2 Methods and analysis

### 2.1 Systematic review study design

This mixed-methods systematic review will be conducted in accordance with PRISMA guidelines. It has been registered with PROSPERO (CRD42020184834) [26].

### 2.2 Systematic review theoretical framework

Our study is conceptually informed by the framework for integrating health into urban and territorial planning which was co-developed by the World Health Organization and UN-Habitat [27]. Informed by this tool, we aim to capture details on multisectoral interventions by setting (e.g., public spaces, movement corridors, cycling paths and active transport); by outcome (e.g., physical activity, access to health care); by principle (e.g., reducing road danger, life-course strategies); and by sector (e.g., housing, economy, etc). We have decided to focus on the dimensions (setting, sector, principle, outcome) while leaving specific details open (e.g., housing as a specific sector needed for multisectoral partnerships). We envisage, for example, that there may be additional sectors found in each country, or that sectors which we expect to be included may not be. In either case, this would be informative for future intervention and policy planning.

### 2.3 Study eligibility criteria

#### 2.3.1 Types of studies

In generating evidence that is relevant and useful to decision-makers and communities, it is valuable to integrate findings from diverse types of research [28]. Mixed-methods systematic reviews integrate qualitative and quantitative research findings and enhance their utility and impact in influencing policy and practice [28]. They also help to better represent the experiences of the communities the research aims to serve. This systematic review will therefore apply a mixed-methods approach, with mixing occurring in three steps: i) extracting both qualitative and quantitative findings, ii) analysing findings, and iii) integrating findings [28].

#### 2.3.2 Inclusion criteria

We will systematically synthesise existing primary quantitative and qualitative evidence in academic and grey literature on intersectoral initiatives for NCD prevention in Africa. We will include all quantitative and qualitative study designs that describe any initiatives to address NCD risk factors interventions, and those that describe associations between such interventions and process, wellbeing, or access-related outcomes. We will also include health service interventions that have an intersectoral component and are focused on NCD prevention. We will focus on multisectoral interventions specifically targeting the prevention of NCDs e.g., cardiovascular disease, or the risk factors for non-communicable diseases e.g., diet and physical activity. This would exclude interventions focused on managing existing NCDs (e.g., interventions to manage disability due to stroke), clinical interventions addressing NCD prevention that do not involve any partnerships (e.g., hospital-based interventions) as well as interventions that may include NCD prevention but do not explicitly say so (e.g., broader water and sanitation (WASH) interventions). Studies must have been conducted in urban settings in African countries, published in the past 30 years, and contain primary or secondary data as well as an analysis of these data. We have chosen this period as it represents the start of the active global promotion of the concept of healthy cities [29]. While we will exclude literature reviews, commentaries, opinion pieces and narrative overviews that do not describe multi-sectoral, health-promoting or preventive health service interventions, we will use these to provide context, background information, point to primary literature and look for cues on identifying grey literature.

### 2.4 Intervention/exposure

We will characterise these intersectoral initiatives using the following features:

i) A focus on an urban area, as indicated by administrative, political and or geographic boundaries [25].
ii) A focus on improving the built and natural environment in urban (including informal) settings. Examples include initiatives to improve neighbourhood walkability, urban green spaces, air or water quality.
iii) A focus on building partnerships across sectors to address the health and wellbeing of the population [1]. Examples include collaborations between grassroots organisations and the local government to improve access to housing, as well as partnerships between agriculture and health to improve nutrition for low-income urban residents. We will also note any enabling legal or policy frameworks which were employed in facilitating these partnerships [30].
iv) A focus on the social infrastructure, participation and empowerment of community members [1]. Examples include initiatives to improve accountability of local governments for delivery of services that influence health, or initiatives to harness social capital for neighbourhood development.
v) A focus on equity in involvement, access and impact of the initiative [1]. Examples include initiatives to assess intra-urban differences in the health outcomes of urban exposures e.g., air pollution and to design targeted interventions for high-risk populations.
vi) Considerations for planetary health. We will note any considerations for planetary health in the design, funding, partnership development, advocacy for, implementation and evaluation of identified intersectoral initiatives.

#### 2.4.1 Types of participants

We will not limit the age, gender, ethnicity of populations targeted by studies included in this systematic review. All study settings and initiatives run by any sectors e.g., education, environment, and health will be included as long as they involve intersectoral collaborations, address NCD risk factors multisectorally and were implemented in African cities.

#### 2.4.2 Outcomes

Given the multiplicity of outcomes of healthy city interventions, we will categorise these according to the following dimensions:

i) Behaviours – examples include increased physical activity, healthier diets, improved stress management, social cohesion, etc.
ii) Built and social environments– examples include increases in the number of sidewalks, street lighting and signage, air and water quality.
iii) Improvement in access to services – examples include improved access to waste management services, more affordable housing, access to healthy foods.
iv) Participation of the population – examples include improved representation of informal residents in the local government.
v) Health profiles and disease outcomes such as obesity, diabetes, hypertension, etc.
vi) Health equity-examples include reduction in diabetes incidence, increase in physical activity participation, improved diabetes outcomes among the urban poor.
vii) The financing, regulation, and implementation of intersectoral collaborations. These include collaborations between two or more of the following players: government agencies, policymakers, the private sector, informal organisations, grassroots organisations, non-profit organisations, multilateral agencies.

### 2.5 Literature search

#### 2.5.1 Electronic bibliographic databases

Literature search strategies will be developed using medical subject headings and text words. We will enlist the help of a medical librarian in developing a search strategy in PubMed using both text words and medical subject headings. The search terms have been included in the appendix of this paper. We will adapt this search to Global Health, SCOPUS, and Web of Science and will include all languages, using the expertise of the research team and collaborating institutions for translation. We expect that most articles will be published in English, French, Spanish, Portuguese, or Arabic, as these languages are frequently spoken in African countries.

We will also conduct a grey literature search using Google to identify reports from local, regional, and international agencies with a focus on healthy city initiatives. We will narrow our search by country, domain and file type and search the first five pages of the references we retrieve. We will also source grey literature from international organisations such as the WHO and UN-Habitat, UNICEF's Child-Friendly Cities Initiative, Partnership for Healthy Cities, WHO Alliance for Healthy Cities, the African Centre for Cities, as well as grey literature databases such as Greynet and Opengrey. We will inform stakeholders across the GDAR network of our initial findings and work with them to identify sources of grey literature and ensure our search is exhaustive. We will also approach key contacts in government and non-governmental agencies, academic topic experts, and our multilateral partners.

#### 2.5.2 Study screening and selection

We will export our search results into EndNote and remove duplicates while uploading them into Covidence [31], a digital systematic review platform, for further review, extraction, and quality assessment. We will assemble a team of researchers across the GDAR network to support the review of the evidence and screen the titles and abstracts against the inclusion and exclusion criteria identified by the study team. Researchers will work in pairs to screen at least 10% of the total number of articles, to assess the percent agreement. The inclusion and exclusion criteria, including definitions of interventions/exposures and outcomes, will be reviewed, clarified, and refined until there is at least 90% agreement across the teams. At this point, all the records will be distributed across the researchers to complete the process individually.

#### 2.5.3 Data extraction

We will proceed to full-text extraction for articles that meet the inclusion and exclusion criteria. Paired teams will be assigned full-text articles to review, using a data extraction template piloted by the senior researchers in the first phase of the screening. A senior researcher from the Network will be appointed to review any conflicts arising from the final data extraction. For non-English language studies, group members with the necessary competence will be asked to accept or reject articles using the inclusion or exclusion criteria. The reference list of identified articles will be used to identify additional references for backward and forward reference search.

We will create a standardised template to facilitate the data extraction from the paired teams. The following information will be noted:

● The name of the first author
● The year of publication
● The organisation that carried out the research (e.g., academic, research, non-profit, government)
● Funder of the research (e.g., a foundation, a government grant, a private sector organisation)
● The source of the publication (e.g., a google search, a specified journal, a government statistical website, an international organisation)
● The country, city, and administrative division
● Whether the study was focused on informal settlements
● Whether the study was focused on or specifically included adolescents
● The setting of the intervention (school, market, workplace, community, etc.)
● Funder of the intervention (if different from the funder of the study)
● Key actors in the development, implementation and evaluation of the intervention (private, public, government, etc.)
● The type of evidence (qualitative, quantitative, mixed methods studies, case studies, reports, etc.)
● The study design (cohort, cross-sectional, key informant interviews, etc.)
● The study size or population size reached
● The response rate
● Population demographics (age, sex, location, urban/rural, etc.)
● The intervention exposure(s) (e.g., active transport intervention, coalition building, etc.)
● Whether and how impact was assessed
● The impact of the intervention
● The analytical method used
● A summary of the key findings
● Quality assessment

#### 2.5.4 Assessing study quality

We will use the qualitative checklist and the cohort study checklist of the Critical Appraisal Skills.

Programme (CASP) to appraise the quality of each study included in this review [32] and will modify this checklist to accommodate cross-sectional studies. Many appraisal tools were available [33], but we chose the Critical Appraisal Skills Programme (CASP), because it is the most commonly used template for appraising and synthesising evidence on public health-related topics, and it is recommended by the Cochrane Qualitative and Implementation Methods Group as well as the World Health Organization [34]. The Critical Appraisal Skills Programme (CASP) was also chosen for its propensity to build the capacity of our research team and support the use of our research in shaping future transdisciplinary research and action in similar spaces, as it is designed to be applied in pedagogic, workshop, and educational settings.

Approximately five senior researchers will appraise 20% of the total number of studies. Questions to be captured by the modified template include whether the study was focused in its approach, the suitability of the recruitment of the cohort, the extent to which bias was measured and minimised, the consideration given to confounding in the design and analysis of the study, the implications of the study for practice, the appropriateness of a qualitative approach where used, the appropriateness of the design, recruitment and data collection strategy, the consideration given to positionality of the researcher and participants, the consideration given to ethical issues, as well as the rigour, clarity and value of the findings. Due to the wide variety of disciplines and papers we will consider, we expect to encounter different degrees of robustness of the data at the quality appraisal stage. While we do not intend to exclude studies or apply thresholds based on study quality, we aim to focus more on information from more comprehensive studies in refining our interpretation. We primarily intend to use the appraisal tool to identify and communicate recurring quality issues in the literature to inform the design of future initiatives. This approach parallels that taken in a previous study on gender and socioeconomic dimensions of public space use for transport in urban Africa [35] conducted by the research team.

#### 2.5.5 Analysis

The analysis plan will be finalised after the screening process. We do not anticipate having sufficient information to conduct a quantitative meta-analysis due to the wide range of study designs, analytic units, and methods of assessment. However, we will be informed by the Bradford Hill criteria [36] in exploring the strength, consistency, and plausibility of the studies to infer possibly causal relationships.

In line with this, we aim to use a parallel convergent method [37] to synthesise the parallel strands of our study, as it involves analysing the qualitative and quantitative papers separately and then integrating them. For our study, we have chosen a parallel convergent approach, primarily for its ability to weigh the qualitative and quantitative findings concurrently, compare them and allow them to simultaneously enrich one another [38], as opposed to an exploratory sequential design where a subsequent quantitative phase is used to test tools whose development is informed by qualitative data, or an explanatory sequential design where the qualitative data is used to contextualize the findings of quantitative data. Similarly, a thematic synthesis is valuable for synthesising evidence to inform interventions [37,39], which is why we have chosen this approach to synthesise the evidence gained. This approach is particularly appropriate for integrating data with various categories, such as the information on the location, impact, evaluation and partnerships in our case. The thematic synthesis typically has three steps, namely the data coding, the development of descriptive themes, and the generation of analytic themes, and these will be transformed into appropriate policy and intervention recommendations for the aims of our study. A summary table of the analytical approach is included in the supplementary material.

#### 2.5.6 Patient and public involvement

Given that this study is a systematic review and does not involve primary data collection, it will not have patient and public involvement during the piloting, screening and extraction phases. However, as we anticipate that the findings will highlight opportunities to improve urban health and reduce NCD risk in the cities of interest, we will share these findings with grassroots organisations, governments, international organisations, private sector organisations as well as the general public. Once our findings are generated, we will tailor our PPI activities accordingly.

## 3 Ethics and dissemination

This study will follow the media and publication policies of the GDAR network (GDAR). Knowledge exchange is a core part of the Network and is integrated into ongoing activities. The knowledge exchange strategy for this study will emphasise the needs of the audiences who will benefit from this information, the key points during this study at which it will be vital to involve them, their preferences for receiving this information, and building long term collaborative partnerships to inform future research and the implementation of the key findings in various partner sites. Primary stakeholders for this project include:

1) Researchers involved in the review: We will work collaboratively to build the skills of the researchers for technical guidance during the piloting, data extraction and analysis. At the end of the project, we will explore how the research produced can inform the direction of the Global Public Health research programme at the University of Cambridge, future collaborations with researchers in the GDAR network and other partners, as well as evaluating the communications strategy.
2) Public health researchers: We will identify and contact public health researchers working on urban health and NCD research in Africa. These researchers will also be considered as an audience for the final research findings and are likely to value the evidence gained and gaps in evidence identified.
3) Global organisations working on policy and programs related to urban health and NCD prevention and management in Africa, such as the World Health Organization, UN-Habitat, UN-Habitat, the UN Interagency Task Force on Noncommunicable Diseases Prevention and Control and the NCD Global Coordination Mechanism, will also be considered as an audience for the final research findings and we will seek to target each of these players with results in context-appropriate ways. For example, global organisations may seek to understand how to align development financing with the evidence on what works for improving urban health as identified from our review. Our work may also inform policy priorities and global recommendations, and support urban health organisations to better frame their messaging.
4) Local stakeholders who influence decisions related to urban health and NCD prevention and management in Africa. These include local government authorities, Ministries of Health at the national and global level, grassroots organisations, community leaders and religious groups. We will solicit their input particularly during the grey literature search, and to understand the evidence needs and preferences of various stakeholders. Local organisations may find it valuable to understand evidence-informed levers for urban health and wellbeing which our findings will synthesise.
5) Organisations that influence, finance or execute urban development projects. These include private sector organisations such as urban developers and architects, intergovernmental organisations such as the African Development Bank and China Development Bank, African philanthropists such as Dangote Foundation and the Tony Elumelu Foundation, relevant government arms such as national Ministries of Budget and National Planning. These organisations will also be considered as an audience for the final research findings and we will seek to target each of these players with results in ways that are adapted to their priorities and evidence needs.

## 4 Conclusion

Given the rapid urban transition ongoing in African countries, cost-effective and sustainable approaches to address NCDs and improve population health are needed. This systematic review will generate useful knowledge to inform improved health via multisectoral interventions, which present a sustainable and promising opportunity in African cities. This systematic review protocol makes use of rigorous methods to search, screen, evaluate and synthesise primary research articles, including the use of a CASP checklist to reduce the risk of bias. A potential limitation is the possibility of missing out on knowledge that is not publicly accessible, as well as making translation errors for articles in different languages, despite the thorough approaches used to gather literature for this study. The wide scope of studies included will allow for a breadth of analyses but could also pose a challenge in synthesising findings with disparate designs and outcomes. These challenges will be reduced given the wide range of multidisciplinary databases searched, the diversity of locations represented by the study team which will support the analysis and synthesis, the use of comprehensive grey literature, as well as input from topic experts who will be engaged throughout the review process. The pan-African scope of this study makes it eligible to serve as a regional body of work and a resource to inform future interventions, practices, and policies.

## Author statements

The pilot was developed and implemented by all team members (EM, AB, TM, CE, LM, TO, LF), with assistance from Isla Kuhn, the medical librarian, University of Cambridge. The protocol was initially drafted by EM and TO. Subsequent drafts were commented on by every member of the team, which informed the revisions and the final draft. All authors have approved this submission and declare no competing interests. No ethical approval is required for this study given that no primary data will be collected.

EM, LM, AB, TO are supported by the 10.13039/501100000272National Institute for Health Research (NIHR) (16/137/34) using UK aid from the UK Government to support global health research. The views expressed in this publication are those of the author(s) and not necessarily those of the NIHR or the UK Department of Health and Social Care.

## Conflict of interest

None declared.
