# Supplementary material for: A protocol for a systematic review on intersectoral interventions to reduce non-communicable disease risk factors in African cities
Source: Public Health Pract (Oxf). 2022 Apr 4;3:100251. doi: 10.1016/j.puhip.2022.100251 (PMC9207189; doi:10.1016/j.puhip.2022.100251)
Supplement: Multimedia component 1 [file mmc1.docx]

# **APPENDIX**

**Pilot searches**

Search terms were created in consultation with a librarian as well as team members. Searches were carried out on PubMed and translated for Scopus, Global Health and Web of Science to offer a good breadth of literature on the intersection of public spaces and health in Africa. An initial search was run in August 2019 with a hit of 3853 articles. From this initial term, search terms were refined to specify examples of non-communicable diseases. The final search was run in September 2019 and yielded 5890 articles. After these terms were agreed upon by the study team, they were translated for Web of Science which yielded 20080 articles, Global Health which yielded 8263 articles and Scopus which yielded 7417 articles.

**Table 1: Search strategy for PubMed**

| **Exposure**    ("public health"[MeSH Terms] OR ("public"[All Fields] AND "health"[All Fields]) OR "public health"[All Fields]) OR (("health"[All Fields] AND "systems"[All Fields]) OR "health systems"[All Fields]) OR (healthy[All Fields] AND ("cities"[MeSH Terms] OR "cities"[All Fields])) OR (("health"[MeSH Terms] OR "health"[All Fields]) AND outcomes[All Fields]) OR ("health promotion"[MeSH Terms] OR ("health"[All Fields] AND "promotion"[All Fields]) OR "health promotion"[All Fields]) OR ("health services"[MeSH Terms] OR ("health"[All Fields] AND "services"[All Fields]) OR "health services"[All Fields]) OR (("disease"[MeSH Terms] OR "disease"[All Fields]) AND ("prevention and control"[Subheading] OR ("prevention"[All Fields] AND "control"[All Fields]) OR "prevention and control"[All Fields] OR "prevention"[All Fields])) OR ("disease"[MeSH Terms] OR "disease"[All Fields]) AND "prevention and control"[Subheading] OR ("prevention"[All Fields] AND "control"[All Fields]) OR "prevention and control"[All Fields] OR "control"[All Fields] OR (("disease"[MeSH Terms] OR "disease"[All Fields]) AND reduction[All Fields]) OR (("disease"[MeSH Terms] OR "disease"[All Fields]) AND improvement[All Fields]) | **Outcome**    (("planet"[All Fields] OR "air"[MeSH Terms] OR "air"[All Fields] OR "climate"[MeSH Terms] OR "climate"[All Fields] OR "ecology"[MeSH Terms] OR "ecology"[All Fields] OR "flooding"[All Fields] OR "floods"[MeSH Terms] OR "floods"[All Fields] OR "social environment"[MeSH Terms] OR ("social"[All Fields] AND "environment"[All Fields]) OR "social environment"[All Fields] OR "ecosystem"[MeSH Terms] OR "ecosystem"[All Fields])) OR ((((("asthma"[MeSH Terms] OR "asthma"[All Fields]) OR ("neoplasms"[MeSH Terms] OR "neoplasms"[All Fields] OR "cancer"[All Fields])) OR ("hypertension"[MeSH Terms] OR "hypertension"[All Fields])) OR ("obesity"[MeSH Terms] OR "obesity"[All Fields] OR "heart diseases"[All Fields] OR "heart disease"[All Fields] OR "heart diseases"[MeSH Terms] OR "diabetes mellitus"[MeSH Terms] OR "diabetes"[All Fields] OR "cardiovascular diseases"[MeSH Terms] OR "cardiovascular diseases"[All Fields] OR "cardiovascular disease"[All Fields] OR "noncommunicable diseases"[MeSH Terms] OR "noncommunicable diseases"[All Fields] OR "non communicable disease"[All Fields] OR "chronic disease"[MeSH Terms] OR "chronic disease"[All Fields] OR "chronic diseases"[All Fields] OR "chronic illness"[All Fields] OR "chronically ill"[All Fields] OR "chronic condition"[All Fields] OR "chronic conditions"[All Fields])) OR wellbeing[All Fields] OR "health"[MeSH Terms] OR "health"[All Fields] OR "wellness"[All Fields] OR "mental health"[MeSH Terms] OR ("mental"[All Fields] AND "health"[All Fields]) OR "mental health"[All Fields] OR "walking"[MeSH Terms] OR "walking"[All Fields] AND cycling[All Fields] OR "diet"[MeSH Terms] OR "behaviour"[All Fields] OR "behavior"[MeSH Terms] OR "behavior"[All Fields] OR "diet"[All Fields] OR "dietary"[All Fields] OR "social capital"[MeSH Terms] OR ("social"[All Fields] AND "capital"[All Fields]) OR "social capital"[All Fields] OR "food"[MeSH Terms] OR "food"[All Fields] OR "communicable diseases"[MeSH Terms] OR ("communicable"[All Fields] AND "diseases"[All Fields]) OR "communicable diseases"[All Fields] OR ("infectious"[All Fields] AND "disease"[All Fields]) OR "infectious disease"[All Fields]) |
| --- | --- |
| **Population**    "ethiopia"[MeSH Terms] OR "ethiopia"[All Fields] OR "gabon"[MeSH Terms] OR "gabon"[All Fields] OR "gambia"[MeSH Terms] OR "gambia"[All Fields] OR "ghana"[MeSH Terms] OR "ghana"[All Fields] OR "guinea"[MeSH Terms] OR "guinea"[All Fields] OR "equatorial guinea"[All Fields] OR "guinea-bissau"[MeSH Terms] OR "guinea-bissau"[All Fields] OR "kenya"[MeSH Terms] OR "kenya"[All Fields] OR "lesotho"[MeSH Terms] OR "lesotho"[All Fields] OR "liberia"[MeSH Terms] OR "liberia"[All Fields] OR "libya"[MeSH Terms] OR "libya"[All Fields] OR "madagascar"[MeSH Terms] OR "madagascar"[All Fields] OR "malawi"[MeSH Terms] OR "malawi"[All Fields] OR "mali"[MeSH Terms] OR "mali"[All Fields] OR "mauritania"[MeSH Terms] OR "mauritania"[All Fields] OR "mauritius"[MeSH Terms] OR "mauritius"[All Fields] OR "morocco"[MeSH Terms] OR "morocco"[All Fields] OR "mozambique"[MeSH Terms] OR "mozambique"[All Fields] OR  "namibia"[MeSH Terms] OR "namibia"[All Fields] OR "niger"[MeSH Terms] OR "niger"[All Fields] OR "nigeria"[MeSH Terms] OR "nigeria"[All Fields] OR "rwanda"[MeSH Terms] OR "rwanda"[All Fields] OR "sao tome and principe"[MeSH Terms] OR "sao tome and principe"[All Fields] OR "senegal"[MeSH Terms] OR "senegal"[All Fields] OR "seychelles"[MeSH Terms] OR "seychelles"[All Fields] OR "sierra leone"[MeSH Terms] OR "sierra leone"[All Fields] OR "somalia"[MeSH Terms] OR "somalia"[All Fields] OR "south africa"[MeSH Terms] OR "south africa"[All Fields] OR "south sudan"[MeSH Terms] OR "south sudan"[All Fields] OR "sudan"[MeSH Terms] OR "sudan"[All Fields] OR "tanzania"[MeSH Terms] OR "tanzania"[All Fields] OR "togo"[MeSH Terms] OR "togo"[All Fields] OR "tunisia"[MeSH Terms] OR "tunisia"[All Fields] OR "uganda"[MeSH Terms] OR "uganda"[All Fields] OR "zambia"[MeSH Terms] OR "zambia"[All Fields] OR "zimbabwe"[MeSH Terms] OR "zimbabwe"[All Fields] OR "africa"[MeSH Terms] OR "africa"[All Fields] | ((((((((((((((((("cities"[MeSH Terms] OR "cities"[All Fields]) OR "city"[All Fields]) OR "town"[All Fields]) OR "towns"[All Fields]) OR "urban"[All Fields]) OR "metropolitan"[All Fields]) OR "conurbation"[All Fields]) OR "municipality"[All Fields]) OR "municipalities"[All Fields]) OR "city planning"[MeSH Terms]) OR "urban planning"[All Fields]) OR "metro"[All Fields]) OR "metropolis"[All Fields]) OR ("megacities"[All Fields] OR "megacity"[All Fields])) OR "urban renewal"[MeSH Terms]) OR ("urban"[All Fields] AND "renewal"[All Fields])) OR "urban renewal"[All Fields]) OR ((((((((((((((("urban"[All Fields] OR "urbanicity"[All Fields]) OR "urbanism"[All Fields]) OR "urbanity"[All Fields]) OR "urbanization"[MeSH Terms]) OR "urbanization"[All Fields]) OR "urbanize"[All Fields]) OR "urbanized"[All Fields]) OR "urbanizes"[All Fields]) OR "urbanizing"[All Fields]) ) OR "urbanisation"[All Fields]) OR "urbanise"[All Fields]) OR "urbanised"[All Fields]) OR "urbanises"[All Fields]) OR "urbanising"[All Fields])      **Total hits: 5890 articles** |

**Table 2: Search strategy for Web of Science**

| Exposure  (planet or air or flood* or ecology or “social environment” or climate or ecosystem or environment or hypertens* or obesity or climate or “heart disease*” or asthma or “cardiovascular disease*” or “chronic condition*” or “noncommunicable disease*” or neoplasm* or food or “chronic illness” or “social capital” or diet* or cancer or walking or cycl* or “communicable disease*” or infectio*) | Outcome    OR (“public health” or “health system*” or “healthy cities’ or “health promotion” or “health service*” or “disease prevention” or “disease control” or “disease reduction” or “disease improvement”) |
| --- | --- |
| Population  AND (city* or cities* or town* or urban* or municipal* or megacity* or megacities or metropol* or conurbation* or town) | AND (ethiopia or gabon or gambia or ghana or guinea or “guinea bissau” or kenya or lesotho or liberia or libya or madagascar or malawi or mauritania or morocco or mauritius or mali or morocco or mozambique or namibia or niger or nigeria or rwanda, or “sao tome and principe” or senegal or seychelles or “sierra leone” or somalia or “south africa” or “south sudan” or tanzania or togo or uganda or zambia or zimbabwer or “cote d’ivoire” or eswatini or swaziland or algeria orbenin or togo or angola or “burkina faso” or botswana or burundi or djibouti or eritrea or sahrawi or burundi or egypt or djibouti or “democractic republic of congo” or comoros or “cape verde” or “central african republic” or chad or africa )  Total hits: 21080 |

**Table 3: Search strategy for Global Health**

| Exposure  (planet OR air OR climate OR ecology OR (floods or flooding or flood) OR social environment OR ecosystem OR asthma OR neoplasms OR cancer OR hypertension OR obesity) | Outcome  ((public health OR health systems OR healthy cities OR health outcomes OR health promotion OR health services OR (disease prevention and control) OR disease reduction OR disease improvement) OR (heart disease OR diabetes mellitus OR diabetes OR ecology OR cardiovascular disease OR noncommunicable diseases OR noncommunicable disease OR cardiovascular diseases OR chronic disease OR chronic diseases OR chronic illness OR chronically ill) OR (chronic condition OR chronic conditions OR wellbeing OR health OR wellness OR mental health OR walking OR cycling OR diet OR dietary OR social capital OR food) OR (communicable diseases OR infectious disease) |
| --- | --- |
| Population  (cities or urban or city ) OR (town or towns) OR metropolitan OR conurbation OR municipality OR municipalities OR urbanization Or city planning OR urban planning OR metro OR metropolis OR megacity | ( ethiopia or gabon or gambia or ghana or guinea or equatorial guinea or guinea-bissau or kenya or lesotho or liberia or libya or madagascar or malawi or mali or mauritania or mauritius or morocco or mozambique or namibia or niger or nigeria or rwanda or sao tome and principe or senegal or seychelles or sierra leone or somalia or south africa or south sudan or sudan or tanzania or togo or tunisia or uganda or zambia or zimbabwe or africa )  Total hits: 8263 |

**Table 4: Search strategy for Scopus**

| Exposure  ( TITLE-ABS-KEY (planet OR air OR climate Or ecology OR “social environment” OR flooding OR floods OR ecosystem OR neoplasms OR asthma OR cancer OR hypertension OR obesity Or “heart diseases” OR “heart disease” OR cardiovascular diseases” OR “cardiovascular disease” ) ) | Outcome  OR ( TITLE-ABS-KEY (“noncommunicable disease”* OR “chronic disease”* OR “chronic illness” OR “chronic conditions” Or wellbeing OR health OR wellness Or “mental health” OR walk* OR cycl* OR behaviour OR behavior OR diet* OR “social capital” OR food OR “communicable disease*” OR “infectious disease*” OR planet OR air OR climate OR ecology OR “social environment” OR flood* OR ecosystem OR neoplasm Or asthma* OR cancer OR hypertens* OR obes* OR “heart disease*” OR “cardiovascular disease*” ) ) OR ( TITLE-ABS-KEY(“public health” OR “health systems*” OR “healthy cities” OR “health outcomes” OR “health promotion” OR “health service*” OR “disease prevention and control” OR “disease reduction” OR “disease improvement”) |
| --- | --- |
| Population  ( TITLE-ABS-KEY ( ethiopia Or gabon Or gambia OR ghana OR guinea OR “Guinea Bissau” OR kenya OR libya OR lesotho OR liberia OR malawi Or madagascar Or mali Or mauritania OR mauritius OR morocco OR mozambique OR namibia OR niger OR nigeria OR rwanda Or “SAO TOME AND PRINCIPE” OR sengal OR seychelles Or senegal OR “SIERRA LEONE” OR somalia OR “SOUTH AFRICA” OR “SOUTH SUDAN” OR sudan OR tanzania OR tunisia OR uganda OR zambia OR zimbabwe OR africa OR “COTE D’IVOIRE” OR “IVORU COAST” OR eswatini OR algeria OR swaziland OR benin OR “BENIN REPUBLIC” OR angola OR botswana OR “BURKINA FASO” OR eritrea OR sahrawii OR burundi OR egypt OR djibouti OR “DEMOCRATIC REPUBLIC OF CONGO” OR comoros OR “CABO VERDE” OR “CAPE VERDE” OR “CENTRAL AFRICAN REPUBLIC” OR “CHAD” OR africa ) ) | (TITLE-ABS-KEY ( cities* OR city* OR town* OR metropol* OR conurbation* OR municipal* OR urban* OR megacity* OR megacities))    Total hits: 7417 |
