# Supplementary material for: A protocol for a systematic review on intersectoral interventions to reduce non-communicable disease risk factors in African cities
Source: Public Health Pract (Oxf). 2022 Apr 4;3:100251. doi: 10.1016/j.puhip.2022.100251 (PMC9207189; doi:10.1016/j.puhip.2022.100251)
Supplement: Multimedia component 2 [file mmc2.docx]

**Intersectoral interventions to address non-communicable disease risk factors in urban Africa: a systematic review**

**Data extraction template**

**May 2020**

| **Field** | **Description** |
| --- | --- |
| **Identification** |  |
| Urban or rural | Was this project in an urban or rural setting? If rural, stop here and recommend for exclusion |
| Sponsorship source | Enter the institution that sponsored the study e.g. National Institutes of Health Research |
| Date of data collection | Enter the start and end year of data collection  *Examples*  2014  2014-2015 (one discrete period of data collection)  2014, 2017 (multiple periods of data collection e.g. cohort)  2015-2015, 2017-2018 (multiple periods of data collection, each spanning multiple years) |
| Date(s) of project implementation | *Examples*  2014  2014-2015 (one discrete period of data collection)  2014, 2017 (multiple periods of data collection e.g. cohort)  2015-2015, 2017-2018 (multiple periods of data collection, each spanning multiple years) |
| Date of publication | Enter the year that the study was published  *Example*  2014 |
| **Author’s contact details** |  |
| Name of the author | Enter the surname and initials of the first author  *Example*  Oyeyemi, AL |
| Authors’ institutional affiliation | Enter the institutional affiliation of each of the authors, with the country of affiliation in brackets  If more than one, list separated by a comma  *Example*  University of Maiduguri (Nigeria), University of Cape Coast (Ghana) |
| Email address of the corresponding author | Enter the email address of the first author or ‘email not provided’ where this is not available  *Example*  andreowerneck@gmail.com |
| Country of affiliation of the first author | Enter the country or countries of the institution(s) where the first author has an affiliation, separating them with a comma whee multiple  *Example*  Kenya, South Africa |
| Country of affiliation of the last author | Enter the country where the last author’s institution or affiliation is located  If more than one, list the country of the first affiliation  If the study has only one author, enter ‘no last author’  *Example*  United States of America |
| Data source | What categories of people were involved in gathering the knowledge generated or information described in this project?  Select all of the following that apply   - Academic organizations e.g. the University of Lagos, University of Nsukka - Research Institute/Centre e.g. the African Population and Health Research Center, Alex Ekwueme center for interdisciplinary research - Government e.g. Government of Ekiti State, community health workers in Awka South local government area - Private sector e.g. Julius Berger, Dangote - International non-profit organisation e.g. the Gates Foundation, Tony Elumelu Foundation, - Regional non-profit organization e.g. the African Union, the Economic Community of West African States - Local non-profit organization or civil society group e.g. Alabiamo Foundation, Mentally Aware Nigeria - Community groups or their representatives e.g Obuwanne age grade, Amawbia; residents of Jakande Estate - Other |
| Specific name of the data source | Enter the specific source of the data  e.g. Anambra State Ministry of Education |
| **About the intervention** |  |
| Country of implementation or planned implementation | Enter the name of the country where the intervention was implemented or will be implemented, separating multiple countries with a comma e.g. Ghana, Nigeria |
| What type of initiative is this? | Select all that apply   - planning a project - forming a collaboration - implementing an intervention - measuring impact - monitoring or evaluation |
| Status of the project | Select one of the following as it relates to your answer above   - the paper describes the plan for the initiative - the paper describes the actual initiative |
| Partner(s) involved in designing the project | Select all of the following that apply   - Academic organizations e.g. the University of Lagos, University of Nsukka - Research Institute/Centre e.g. the African Population and Health Research Center, Alex Ekwueme center for interdisciplinary research - Government e.g. Government of Ekiti State, community health workers in Awka South local government area - Private sector e.g. Julius Berger, Dangote - International non-profit organisation e.g. the Gates Foundation, Tony Elumelu Foundation, - Regional non-profit organization e.g. the African Union, the Economic Community of West African States - Local non-profit organization or civil society group e.g. Alabiamo Foundation, Mentally Aware Nigeria - Community groups or their representatives e.g Obuwanne age grade, Amawbia; residents of Jakande Estate - Other |
| Name of the partner(s) involved in designing the project | Write down the name of partner(s) involved in designing the project. Separate multiple partners with a comma e.g Johnson and Johnson, Dangote Foundation |
| Sector(s) involved in designing the intervention | This refers to the sectors that the multisectoral partners you just mentioned above represent. Separate multiple entries with a comma  e.g transport, health, governance |
| Planetary health considerations in project design | What planetary health considerations were given to the design of the project? Planetary health concerns the health impacts of disruptions in the earth’s natural systems.  e.g. when evaluating the bids from potential implementers, the government gave more priority to |
| Partner(s) involved in funding the project | This includes not only the sponsor of the paper which you noted in the beginning, but also the funder(s) of every cycle of the project - the design, implementation and evaluation.  Select all of the following that apply.   - Academic organizations e.g. the University of Lagos, University of Nsukka - Research Institute/Centre e.g. the African Population and Health Research Center, Alex Ekwueme center for interdisciplinary research - Government e.g. Government of Ekiti State, community health workers in Awka South local government area - Private sector e.g. Julius Berger, Dangote - International non-profit organisation e.g. the Gates Foundation, Tony Elumelu Foundation, - Regional non-profit organization e.g. the African Union, the Economic Community of West African States - Local non-profit organization or civil society group e.g. Alabiamo Foundation, Mentally Aware Nigeria - Community groups or their representatives e.g Obuwanne age grade, Amawbia; residents of Jakande Estate - Other |
| Name of the partner(s) involved in funding the project | Write down the name of partner(s) involved in funding the project. Separate multiple partners with a comma e.g Johnson and Johnson, Dangote Foundation |
| Sector(s) involved in funding the intervention | This refers to the sectors that the multisectoral partners you just mentioned above represent, Separate multiple entries with a comma  e.g transport, health, governance |
| Planetary health considerations in project funding | What planetary health considerations were given to funding the project? Planetary health concerns the health impacts of disruptions in the earth’s natural systems.  e.g. the government awarded the contract for the project to the bidder with the best environmental footprint |
| Partner(s) responsible for implementing the project | Select all of the following that apply   - Academic organizations e.g. the University of Lagos, University of Nsukka - Research Institute/Centre e.g. the African Population and Health Research Center, Alex Ekwueme center for interdisciplinary research - Government e.g. Government of Ekiti State, community health workers in Awka South local government area - Private sector e.g. Julius Berger, Dangote - International non-profit organisation e.g. the Gates Foundation, Tony Elumelu Foundation, - Regional non-profit organization e.g. the African Union, the Economic Community of West African States - Local non-profit organization or civil society group e.g. Alabiamo Foundation, Mentally Aware Nigeria - Community groups or their representatives e.g Obuwanne age grade, Amawbia; residents of Jakande Estate - Other |
| Name of the partner(s) involved in implementing the project | Write down the name of partner(s) involved in implementing the project. Separate multiple partners with a comma e.g Johnson and Johnson, Dangote Foundation |
| Sector(s) involved in implementing the intervention | This refers to the sectors that the multisectoral partners you just mentioned above represent, Separate multiple entries with a comma  e.g transport, health, governance |
| Planetary health considerations in project implementation | What planetary health considerations were given to the implementation of the project? Planetary health concerns the health impacts of disruptions in the earth’s natural systems.  e.g. project resources were sourced locally to minimize the environmental imprint from air and road transportation |
| Partner(s) involved in evaluating the project | Select all of the following that apply   - Academic organizations e.g. the University of Lagos, University of Nsukka - Research Institute/Centre e.g. the African Population and Health Research Center, Alex Ekwueme center for interdisciplinary research - Government e.g. Government of Ekiti State, community health workers in Awka South local government area - Private sector e.g. Julius Berger, Dangote - International non-profit organisation e.g. the Gates Foundation, Tony Elumelu Foundation, - Regional non-profit organization e.g. the African Union, the Economic Community of West African States - Local non-profit organization or civil society group e.g. Alabiamo Foundation, Mentally Aware Nigeria - Community groups or their representatives e.g Obuwanne age grade, Amawbia; residents of Jakande Estate - Other |
| Name of the partner(s) involved in evaluating the project | Write down the name of partner(s) involved in evaluating the project. Separate multiple partners with a comma e.g Johnson and Johnson, Dangote Foundation |
| Sector(s) involved in evaluating the intervention | This refers to the sectors that the multisectoral partners you just mentioned above represent, Separate multiple entries with a comma  e.g transport, health, governance |
| Planetary health considerations in project evaluation | What planetary health considerations were given to the evaluation of the project? Planetary health concerns the health impacts of disruptions in the earth’s natural systems.  e.g. an environmental impact assessment was included in the evaluation of the impact of the construction project |
| Partner(s) involved in advocating for the project | Select all of the following that apply   - Academic organizations e.g. the University of Lagos, University of Nsukka - Research Institute/Centre e.g. the African Population and Health Research Center, Alex Ekwueme center for interdisciplinary research - Government e.g. Government of Ekiti State, community health workers in Awka South local government area - Private sector e.g. Julius Berger, Dangote - International non-profit organisation e.g. the Gates Foundation, Tony Elumelu Foundation, - Regional non-profit organization e.g. the African Union, the Economic Community of West African States - Local non-profit organization or civil society group e.g. Alabiamo Foundation, Mentally Aware Nigeria - Community groups or their representatives e.g Obuwanne age grade, Amawbia; residents of Jakande Estate - Other |
| Name of the partner(s) involved in advocating the project | Write down the name of partner(s) involved in advocating the project. Separate multiple partners with a comma e.g Johnson and Johnson, Dangote Foundation |
| Sector(s) involved in advocating for the intervention | This refers to the sectors that the multisectoral partners you just mentioned above represent, Separate multiple entries with a comma  e.g transport, health, governance |
| Planetary health considerations in project advocacy | What planetary health considerations were given to advocacy for the project? Planetary health concerns the health impacts of disruptions in the earth’s natural systems.  e.g. the community requested the project because of the high rates of pollution in their town |
| Entrypoint driving the project | As you read the article what was the entry point that drove the project?  Select as many as are applicable  The setting e.g. public spaces, streets  The sector e.g housing, health  The principle e.g. healthy cities, road danger reduction  The outcome e.g. improving air quality, improving physical activity |
| Principle guiding implementation | This is the underlying justification for the project. Add as many as you think of.  For example, a project that aimed to improve health outcomes for the urban poor, will have its guiding principle as health equity.  Other examples include but are not limited to equity, social justice, healthy cities, inclusive participation, healthy urban governance, social and environmental justice, road danger reduction, community action, age friendly places, healthy cities etc |
| Country where the intervention was implemented | Separate multiple entries with a comma  e.g. Kenya, Nigeria |
| State or province where the intervention was implemented | Separate multiple entries with a comma  e.g. Lagos, Bungoma county |
| Town or locality | Separate multiple entries with a comma  e.g. Agege, Webuye municipality |
| Setting(s) of the intervention | This refers to where the intervention was implemented. Separate multiple entries with a comma  e.g parks, the home, the school, church, mosque, marketplace |
| Informality or formality of the setting | Note the nature of the setting(s). Select both if applicable  Formal  Informal  Not mentioned |
| Outcome of the intervention | What was the desired or measured outcome of the intervention?  Choose as many as apply, specifying the particular outcome in brackets  Health behaviours  Health outcomes  Developing collaborations  Improving the built environment  Improving the natural environment  Providing social infrastructure to improve participation and empowerment of community members e.g creation of a community group to support the mental health of teenagers  Access to services and physical infrastructure e.g. providing free library cards for mothers |
| Specify the outcome(s) | Separate multiple with a comma  e.g improved physical activity, reduced diabetes, developing a partnership between local governments and the Ministry of Health, creating sidewalks on roads in a community, greening a neighbourhood |
| What population was the intervention targeted at? | Separate multiple with a comma  e.g. pregnant women in Sogunro community |
| Outcome measurement | Was the outcome measured?  Yes  No |
| Outcome method | If your answer above was yes, enter used to assess the outcomes:  · Subjective (Usually this means the participant has self-reported)  · Objective (Observed by the researcher. For example, counting people using particular travel modes)  · Both subjective and objective |
| Outcome method specification | Specify how the outcome method was measured  e.g. through researcher led observation of weekly physical activity at the new park |
| Analysis method - quantitative | Enter a small amount of free text summarising the analysis methods. For quantitative studies it is important to distinguish between basic statistical methods for describing quantities of variables (e.g. modes, means, standard deviations), assessing relationships (e.g. correlations) and those that account for potential confounding factors (e.g. multivariable regression modelling).    For quantitative or mixed method studies, complete this field. Otherwise (i.e. for qualitative studies) enter ‘not applicable’ |
| Analysis method - qualitative | Enter a small amount of free text summarising the analysis methods, e.g. thematic analysis.    For qualitative or mixed method studies, complete this field. Otherwise (i.e. for quantitative studies) enter ‘not applicable’ |
| Facilitators and barriers | Note any facilitators and barriers to design, implementation, funding and advocacy for the project  e.g. facilitators - religious groups contributed in advocating for the project at the local government level  barriers - funding limitations made it impossible to complete the project  barriers - a change in government led to a redesign of the original project plan  facilitator – evidence was useful in convincing policy makers to integrate the intervention into the health system |
| Sustainability considerations | While the previous entry details the short term implementation of the project, this entry concerns the considerations for the long-term sustainability mentioned in the article.  For example, these could be policies, procedures and activities put in place to ensure the project could go on long term, or challenges faced that could limit the project’s continuity, etc |
| Lessons learned | Note any other lessons learned throughout the cycle of the project - from its design, implementation, evaluation and advocacy for the project, that you think could inform future replication even though they do not fall under the barriers and facilitators listed above  e.g. Although the previous intervention may have contributed to the good knowledge about TB and care-seeking attitudes displayed by respondents in the communities,sustaining active case finding through public–private partnership can go a long way to reduce TB burden, especially in rural communities where healthcare systems are gener-ally weak or inadequate. Adequate funding of TB control activities is critical in eliminating TB as a public health problem, and the private sector participation such as this is a welcome development |
| **Study methods** |  |
| Research type | Enter the research type:  Use ONE of the following categories:   - Quantitative (measures of values or counts expressed as numbers e.g. the data generated from surveys, questionnaires, measurements) - Qualitative (description of phenomena often expressed as text e.g. interviews, focus groups) - Mixed method (Both quantitative and qualitative methods used) |
| Study design - quantitative | Enter the study design.  Quantitative or mixed method study - use ONE of the following categories:   - Randomised controlled trial - Cohort study - Case-control study - Cross sectional study - Case report / Case study - Other – please, specify   For quantitative or mixed method studies, complete this field. Otherwise (i.e. for qualitative studies) enter ‘not applicable’ |
| Study design - qualitative | Enter the study design.  Qualitative or mixed method study – use ONE of the following categories:   - Grounded theory - Case study - Historical / Narratives - Participatory research / Action research - Phenomenology - Ethnography / Observation - Other – please, specify   Ethnography: Immersion of the researcher the participants’ environment, typically through observation (note, do not confuse this design with observational quantitative designs like traffic counting)  For qualitative or mixed method studies, complete this field. Otherwise (i.e. for quantitative studies) enter ‘not applicable’ |
| Study method – quantitative | If the study includes a quantitative element, enter the method type.  Enter a small amount of free text summarising the study methods. This can usually be cut and pasted from the abstract.  *Examples*  Two-day vox pop survey, structured questionnaire at two main trip destinations  STEPs survey  Household survey and interview  For quantitative or mixed method studies, complete this field. Otherwise (i.e. for qualitative studies) enter ‘not applicable’ |
| Study method - qualitative | If the study includes a qualitative element, enter the method type.  Use the following categories:  If more than one, list separated by a comma   - Structured – interview , survey, questionnaire - Un-structured or Semi structured - interview , survey, questionnaire - In depth- interview / Key informants - Focus groups / Group discussions - Field Notes - Narrative descriptions - Audio tapes - Video tapes - Seasonal calendars - Transect walks - Participatory mapping / Modelling - Other– please, enter a small amount of free text summarising the study methods.   For qualitative or mixed method studies, complete this field. Otherwise (i.e. for quantitative studies) enter ‘not applicable’ |
| Exposure category | Enter ALL of the categories of exposure that were targeted in the intervention  Use the following categories:  If more than one, list separated by a comma   - Individual characteristics e.g age, sex - Interpersonal characteristics e.g. - Built environment - Natural environment - Socio-cultural environment - Policy or wider environment   . |
| Inclusion criteria | Enter a small amount of free text summarising the types of participants included in the intervention. This can usually be cut and pasted from the article.    *Example*  Residents in 34 communities who had symptoms of TB |
| Exclusion criteria | Enter a small amount of free text summarising the types of participants excluded from the intervention. This can usually be cut and pasted from the article.    In many cases, there will be no explicit exclusion criteria listed. If this is the case, enter ‘not reported’.    *Example*  Participants with a diagnosis of cancer were excluded from the intervention |
| Group differences | Enter a small amount of free text summarising whether there were any differences between participant groups at baseline. This only applies to studies that used a controlled experimental design (and it is likely that the majority of literature identified will not use this type of design). This can usually be cut and pasted from the article.    If this does not apply, enter ‘not applicable’. |
| Additional population data |  |
| Sample size | Enter a small amount of free text describing the sample size    *Examples*  10,128 individuals  100 households, 1 member of each household  Ouagadougou - 754 households, 3682 individuals, Bamako - 251 households, 1666 individuals |
| Response rate | Enter a small amount of free text describing the response rate, if provided. If reported, this is typically expressed as a percentage. It is likely that many studies will not provide this information.    If not reported, enter ‘not reported’    *Example*  78% response rate |
| Age | Enter a small amount of free text describing the age of participants. If possible, enter this in the format ‘mean or median (standard deviation)’. Other common reporting formats are a range, or the proportion of participants that fall within a particular age range.    If not reported, enter ‘not reported’    *Examples*  45.3 (2.1) years  15-60 years  48% of participants were aged 30-44 years  Over 13 years |
| Sex | Enter a small amount of free text describing the sex of participants. If possible, enter this in the form ‘number (percentage)’    If not reported, enter ‘not reported’    *Examples*  100 (25%) female; 300 (75%) male  55% female  Female only |
| Direction of relationships | Enter a small amount of free text summarising the direction of the relationships found between exposures and outcomes. Try to keep this as succinct as possible, although it is likely that some studies will report a large number of relationships. Do not worry about reporting the size of the relationship.    *Examples*  Communities with pipe borne water had less cases of cholera |
| Main idea | Imagine you would like to summarise the article to a colleague in few sentences. What would you tell him/her?  Try to get the main idea of the article. Limit your answer to 1 paragraph containing 3 to 5 sentences (maximum) |
