# Supplementary material for: A protocol for a systematic review on intersectoral interventions to reduce non-communicable disease risk factors in African cities
Source: Public Health Pract (Oxf). 2022 Apr 4;3:100251. doi: 10.1016/j.puhip.2022.100251 (PMC9207189; doi:10.1016/j.puhip.2022.100251)
Supplement: Multimedia component 3 [file mmc3.docx]

| **Step** | **Data** | **Description** | **Key questions to explore** | **Output** |
| --- | --- | --- | --- | --- |
| 2^nd^ order analysis (coding) | Extracted data  Quantitative data | Coding of qualitative data  Descriptive summary tables of quantitative data | Key extraction domains as per the extraction tool (available in the appendix)  CASP tools (available in the appendix) | Descriptive codes |
| 2^nd^ order analysis (Translation) | Extracted data  Quantitative data | Translation of the codes into themes  Creating of a joint display table with the key themes and quantitative output for side-by-side analysis | Components, partners, challenges, opportunities and outcomes of multisectoral initiatives  Identification of quality issues for future knowledge co-production | Overarching themes |
| 3^rd^ order analysis (Transformation) | Extracted data  Quantitative data  Grey literature | Transformation of the codes into priorities into policy & intervention recommendations | What are the implications of the research findings for future policy and interventions to address non-communicable diseases via multisectoral initiatives in Africa? | Provisional recommendations for policy, action and future transdisciplinary research |
| Validation of the emergent priorities | Extracted data  Quantitative data  Grey literature | Input from research steering group members involving:  i) Presentation of analytic approach  ii) Presentation of the overarching themes  iii) Presentation of transformed data  iv) Invitation for comments which may agree, disagree, expound on, or add emergent considerations that need to be captured | What are the implications of the research findings for future policy and interventions to address non-communicable diseases via multisectoral initiatives in Africa? | Finalized recommendations for policy, action and future transdisciplinary research |
