# Supplementary material for: A protocol for a systematic review on intersectoral interventions to reduce non-communicable disease risk factors in African cities
Source: Public Health Pract (Oxf). 2022 Apr 4;3:100251. doi: 10.1016/j.puhip.2022.100251 (PMC9207189; doi:10.1016/j.puhip.2022.100251)
Supplement: Multimedia component 4 [file mmc4.docx]

**Modified CASP Cohort Study Checklist (for quantitative studies)**

| Section A: Are the results of the study valid? | |
| --- | --- |
| 1 | Did the study address a clearly focused issue?  *HINT: A question can be ‘focused’ in terms of:*  *● the population studied*  *● the risk factors studied*  *● is it clear whether the study tried to detect a beneficial or harmful effect the outcomes considered*  ☐ Yes  ☐ Can’t tell  ☐ No |
| 2 | Was the cohort/sample recruited in an acceptable way?  *HINT: Look for selection bias which might compromise the generalisability of the findings:*  *● was the cohort/sample representative of a defined population*  *● was there something special about the cohort/sample*  *● was everybody included who should have been*  ☐ Yes  ☐ Can’t tell  ☐ No |
| 3 | Was the exposure accurately measured to minimise bias?  *HINT: Look for measurement or classification bias:*  *● did they use subjective or objective measurements*  *● do the measurements truly reflect what you want them to (have they been validated) ● were all the subjects classified into exposure groups using the same procedure*  ☐ Yes  ☐ Can’t tell  ☐ No |

| 5a | Have the authors identified all important confounding factors?  *HINT: list the ones you think might be important, and ones the author missed*  ☐ Yes  ☐ Can’t tell  ☐ No |
| --- | --- |
| 5b | Have they taken account of the confounding factors in the design and/or analysis?  *HINT: look for restriction in design, and techniques e.g. modelling, stratified-,*  *regression-, or sensitivity analysis to correct, control or adjust for confounding factors*  ☐ Yes  ☐ Can’t tell  ☐ No |
| 6a | For longitudinal studies: Was the follow up of subjects complete enough?  *HINT: Consider:*  *● the good or bad effects should have had long enough to reveal themselves*  *● the persons that are lost to follow-up may have different outcomes than those available for assessment*  *● in an open or dynamic cohort, was there anything special about the outcome of the people leaving, or the exposure of the people entering the cohort*  ☐ Yes  ☐ Can’t tell  ☐ No  ☐ N/A (for cross-sectional studies) |
| 6b | For longitudinal studies: Was the follow up of subjects long enough?  *HINT: Consider:*  *● the good or bad effects should have had long enough to reveal themselves*  *● the persons that are lost to follow-up may have different outcomes than those available for assessment*  *● in an open or dynamic cohort, was there anything special about the outcome of the people leaving, or the exposure of the people entering the cohort*  ☐ Yes  ☐ Can’t tell  ☐ No  ☐ N/A (for cross-sectional studies) |
| Section B: What are the results? | |
| 7 | What are the results of this study?  *HINT: Consider:*  *● what are the bottom-line results*  *● have they reported the rate or the proportion between the exposed/unexposed, the ratio/rate difference*  *● how strong is the association between exposure and outcome (RR)*  *● what is the absolute risk reduction (ARR)*  **Enter your response in the space below** |
| 8 | How precise are the results?  *HINT: look for the range of the confidence intervals, if given*  **Enter your response in the space below** |
| 9 | Do you believe the results?  *HINT: Consider:*  *● big effect is hard to ignore*  *● can it be due to bias, chance or confounding ● are the design and methods of this study sufficiently flawed to make the results unreliable*  *● Bradford Hills criteria (e.g. time sequence, dose-response gradient, biological*  *plausibility, consistency)*  ☐ Yes  ☐ Can’t tell  ☐ No |

| Section C: Will the results help locally? | |
| --- | --- |
| 10 | Can the results be applied to the local population?  *HINT: Consider whether:*  *● a cohort/cross-sectional study was the appropriate method to answer this question ● the subjects covered in this study could be sufficiently different from your population to cause concern*  *● your local setting is likely to differ much from that of the study*  *● you can quantify the local benefits and harms*  ☐ Yes  ☐ Can’t tell  ☐ No |
| 11 | Do the results of this study fit with other available evidence?  ☐ Yes  ☐ Can’t tell  ☐ No |
| 12 | What are the implications of this study for practice?  *HINT: Consider:*  *● one observational study rarely provides sufficiently robust evidence to recommend changes to clinical practice or within health policy decision making*  *● for certain questions, observational studies provide the only evidence*  *● recommendations from observational studies are always stronger when supported by other evidence*  ☐ Yes  ☐ Can’t tell  ☐ No |
